# Supplementary material for: Alcohol use disorders are associated with higher healthcare expenditure among older adults with suspected cognitive impairment: A registry-based cross-sectional study
Source: PLoS One. 2026 Jan 7;21(1):e0340484. doi: 10.1371/journal.pone.0340484 (PMC12779075; doi:10.1371/journal.pone.0340484)
Supplement: S2 Table — (DOCX) [file pone.0340484.s002.docx]

**S2 Table.** **Unadjusted and adjusted linear regressions of the total costs one year before and after NorCog assessment, for alcohol consumption only.**

| **Independent variables** | **One year before assessment** | | | | **One year after assessment** | | | |
| --- | --- | --- | --- | --- | --- | --- | --- | --- |
|  | **Unadjusted model** | | **Adjusted model** | | **Unadjusted model** | | **Adjusted model** | |
|  | **RC (95% CI)** | **p** | **RC (95% CI)** | **p** | **RC (95% CI)** | **p** | **RC (95% CI)** | **p** |
| ***Primary independent variable*** |  |  |  |  |  |  |  |  |
| Alcohol consumption, as reported by next of kin | 0.01 (-0.15; 0.17) | 0.898 | -0.004 (-0.15; 0.14) | 0.962 | -0.11 (-0.29; 0.07) | 0.236 | -0.10 (-0.28; 0.08) | 0.272 |
| Never | 0.16 (0.03; 0.29) | **0.020** | 0.10 (-0.02; 0.22) | 0.119 | 0.10 (-0.04; 0.23) | 0.166 | 0.09 (-0.04; 0.22) | 0.177 |
| Not at all the last year | Reference |  | Reference |  | Reference |  | Reference |  |
| A few times a year | -0.14 (-0.31; 0.03) | 0.103 | -0.02 (-0.18; 0.13) | 0.796 | -0.15 (-0.34; 0.03) | 0.105 | -0.07 (-0.25; 0.10) | 0.416 |
| Once a month | -0.12 (-0.28; 0.04) | 0.129 | -0.04 (-0.19; 0.11) | 0.572 | 0.02 (-0.15; 0.19) | 0.837 | 0.05 (-0.12; 0.21) | 0.586 |
| 2-3 times a month | -0.04 (-0.19; 0.11) | 0.620 | 0.05 (-0.08; 0.19) | 0.446 | -0.02 (-0.18; 0.14) | 0.810 | 0.02 (-0.13; 0.16) | 0.789 |
| Once a week | -0.19 (-0.34; -0.05) | **0.010** | -0.08 (-0.21; 0.06) | 0.272 | -0.02 (-0.16; 0.12) | 0.786 | -0.002 (-0.14; 0.13) | 0.978 |
| 2-3 times a week | -0.04 (-0.18; 0.11) | 0.630 | -0.003 (-0.14; 0.13) | 0.965 | -0.02 (-0.17; 0.13) | 0.767 | -0.03 (-0.17; 0.12) | 0.729 |
| 4-7 times a week | 0.01 (-0.15; 0.17) | 0.898 | -0.004 (-0.15; 0.14) | 0.962 | -0.11 (-0.29; 0.07) | 0.236 | -0.10 (-0.28; 0.08) | 0.272 |
| ***Covariates*** |  |  |  |  |  |  |  |  |
| Age | 0.005 (-0.001; 0.01) | 0.114 | -0.004 (-0.01; 0.002) | 0.205 | -0.01 (-0.02; -0.004) | **0.001** | -0.01 (-0.02; -0.005) | **0.001** |
| Sex, male | 0.13 (0.04; 0.21) | **0.003** | 0.09 (0.01; 0.17) | **0.030** | 0.20 (0.12; 0.29) | **<0.001** | 0.13 (0.04; 0.22) | **0.004** |
| Education (no. of years) | -0.01 (-0.02; 0.0005) | 0.061 | 0.004 (-0.01; 0.02) | 0.534 | 0.005 (-0.007; 0.02) | 0.381 | 0.003 (-0.01; 0.02) | 0.699 |
| Employment status |  |  |  |  |  |  |  |  |
| Not currently working | Reference |  | Reference |  | Reference |  | Reference |  |
| Working 10% or more | -0.30 (-0.52; -0.08) | **0.009** | -0.17 (-0.38; 0.04) | 0.117 | -0.10 (-0.32; 0.13) | 0.409 | -0.15 (-0.38; 0.08) | 0.213 |
| Sick leave/disability benefits | 0.06 (-0.13; 0.26) | 0.522 | 0.02 (-0.17; 0.20) | 0.870 | 0.18 (-0.02; 0.39) | 0.083 | 0.005 (-0.20; 0.20) | 0.965 |
| Retired | -0.03 (-0.12; 0.05) | 0.443 | -0.02 (-0.10; 0.06) | 0.583 | -0.09 (-0.18; -0.0002) | 0.050 | -0.09 (-0.18; -0.006) | **0.037** |
| Receives domiciliary care, Yes | 0.43 (0.35; 0.52) | **<0.001** | 0.29 (0.20; 0.39) | **<0.001** | 0.08 (-0.01; 0.17) | 0.099 | 0.0001 (-0.11; 0.11) | 0.998 |
| Number of chronic diseases | 0.15 (0.13; 0.17) | **<0.001** | 0.07 (0.05; 0.10) | **<0.001** | 0.11 (0.08; 0.13) | **<0.001** | 0.05 (0.03; 0.08) | **<0.001** |
| Number of medications^a^  Number of medications x Number of medications^a^ | 0.18 (0.02)^b^  -0.006 (0.002)^b^ | **<0.001**  **0.002** | 0.13 (0.02)^b^  -0.005 (0.002)^b^ | **<0.001**  **0.005** | 0.08 (0.07; 0.10) | **<0.001** | 0.07 (0.05; 0.08) | **<0.001** |
| Tobacco smoking habits, as reported by next of kin |  |  |  |  |  |  |  |  |
| Never smoked | Reference |  | Reference |  | Reference |  | Reference |  |
| Smoked previously but no longer smokes | -0.02 (-0.10; 0.07) | 0.737 | -0.10 (-0.18; -0.01) | **0.022** | 0.02 (-0.07; 0.11) | 0.616 | -0.07 (-0.17; 0.02) | 0.110 |
| Currently smoking | 0.03 (-0.11; 0.16) | 0.710 | -0.11 (-0.24; 0.02) | 0.112 | -0.07 (-0.20; 0.06) | 0.300 | -0.21 (-0.34; -0.07) | **0.002** |
| MMSE-NR2/3 sum score | 0.01 (0.005; 0.02) | **0.002** | 0.01 (-0.001; 0.02) | 0.067 | 0.03 (0.02; 0.04) | **<0.001** | 0.02 (0.01; 0.03) | **<0.001** |
| NPI-Q subsyndrome scores |  |  |  |  |  |  |  |  |
| NPI-Depression score | 0.03 (0.02; 0.04) | **<0.001** | 0.02 (0.005; 0.03) | **0.008** | 0.02 (0.007; 0.03) | **0.002** | 0.02 (0.0002; 0.03) | **0.047** |
| NPI-Agitation score | 0.03 (0.01; 0.06) | **0.002** | 0.01 (-0.01; 0.03) | 0.401 | 0.02 (0.0003; 0.05) | **0.047** | 0.002 (-0.02; 0.03) | 0.855 |
| NPI-Psychosis score | 0.01 (-0.02; 0.05) | 0.480 | -0.03 (-0.07; 0.01) | 0.183 | 0.02 (-0.01; 0.06) | 0.225 | 0.02 (-0.02; 0.06) | 0.357 |
| PADL sum score | -0.09 (-0.12; -0.07) | **<0.001** | -0.04 (-0.07; -0.01) | **0.009** | -0.03 (-0.06; -0.006) | **0.018** | -0.03 (-0.06; 0.004) | 0.082 |
| Cognitive diagnostic conclusion |  |  |  |  |  |  |  |  |
| Subjective cognitive impairment (SCI) | 0.02 (-0.22; 0.25) | 0.900 | 0.03 (-0.20; 0.26) | 0.791 | 0.01 (-0.20; 0.23) | 0.903 | -0.18 (-0.41; 0.04) | 0.109 |
| Mild cognitive impairment (MCI) | 0.25 (0.16; 0.34) | **<0.001** | 0.25 (0.15; 0.35) | **<0.001** | 0.19 (0.09; 0.28) | **<0.001** | 0.06 (-0.04; 0.16) | 0.212 |
| Dementia | Reference |  | Reference |  | Reference |  | Reference |  |
| “Other diagnoses” | 0.34 (0.20; 0.48) | **<0.001** | 0.31 (0.18; 0.45) | **<0.001** | 0.21 (0.06; 0.36) | **0.005** | 0.07 (-0.08; 0.22) | 0.353 |
| Next of kin’s relationship to participant |  |  |  |  |  |  |  |  |
| Spouse/cohabitant | Reference |  | Reference |  | Reference |  | Reference |  |
| Child/child-in-law | 0.03 (-0.06; 0.11) | 0.520 | -0.09 (-0.18; 0.006) | 0.067 | -0.11 (-0.20; -0.03) | **0.012** | -0.03 (-0.13; 0.08) | 0.594 |
| Other (neighbour, friend, sibling etc.) | 0.19 (0.03; 0.35) | **0.022** | 0.03 (-0.12; 0.18) | 0.703 | 0.03 (-0.14; 0.20) | 0.752 | 0.04 (-0.13; 0.20) | 0.665 |

Outcome values were log-transformed prior to analysis. Significant p values in bold. In the adjusted models, all variables listed were adjusted for. Inverse probability weighting was employed. AUD, Alcohol use disorder; RC, Regression coefficient; CI, Confidence interval; ICD, International Classification of Diseases; MMSE-NR2/3, Mini-Mental State Examination – Norwegian Revised Version 2/3; NPI-Q, The Neuropsychiatric Inventory – Questionnaire; PADL, Personal Activities of Daily Living.

^a^“Number of medications” showed a curvilinear association with the outcome in the adjusted model of healthcare costs one year prior to NorCog.

^b^Standard error instead of CI is presented due to second-order term.
